# Mono-ADP-ribosylation by PARP10 and PARP14 in genome stability

**DOI:** 10.1093/narcan/zcad009

**Published:** 2023-02-20

**Authors:** Ashna Dhoonmoon, Claudia M Nicolae

**Affiliations:** Department of Biochemistry and Molecular Biology, The Pennsylvania State University College of Medicine, Hershey, PA 17033, USA; Department of Biochemistry and Molecular Biology, The Pennsylvania State University College of Medicine, Hershey, PA 17033, USA

## Abstract

ADP-ribosylation is a post-translational modification involved in a variety of processes including DNA damage repair, transcriptional regulation, and cellular proliferation. Depending on the number of ADP moieties transferred to target proteins, ADP-ribosylation can be classified either as mono-ADP-ribosylation (MARylation) or poly-ADP-ribosylation (PARylation). This post-translational modification is catalyzed by enzymes known as ADP-ribosyltransferases (ARTs), which include the poly (ADP-ribose)-polymerase (PARP) superfamily of proteins. Certain members of the PARP family including PARP1 and PARP2 have been extensively studied and assessed as therapeutic targets. However, the other members of the PARP family of protein are not as well studied but have gained attention in recent years given findings suggesting their roles in an increasing number of cellular processes. Among these other members are PARP10 and PARP14, which have gradually emerged as key players in maintenance of genomic stability and carcinogenesis. PARP10 and PARP14 catalyze the transfer of a single ADP moiety to target proteins. Here, we summarize the current knowledge on MARylation in DNA repair and cancer, focusing on PARP10 and PARP14. We highlight the roles of PARP10 and PARP14 in cancer progression and response to chemotherapeutics and briefly discuss currently known PARP10 and PARP14 inhibitors.

## Graphical Abstract

**Graphical Abstract:**
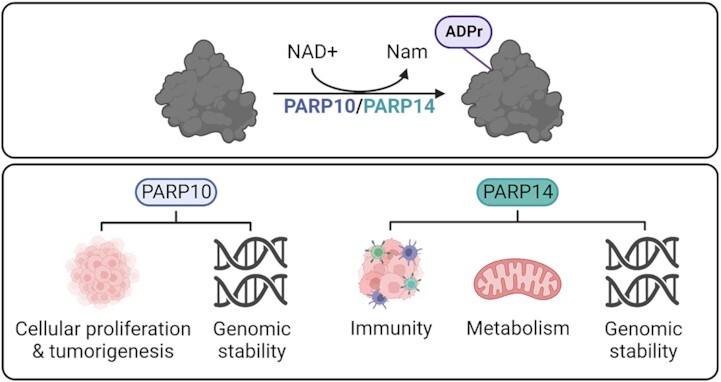
PARP10 and PARP14 catalyze mono-ADP-ribosylation of target proteins to modulate several different biological processes, including cellular proliferation and genomic stability for PARP10, and immunity, metabolism and genomic stability for PARP14.

## INTRODUCTION

### ADP-ribosylation

**Figure 1.**
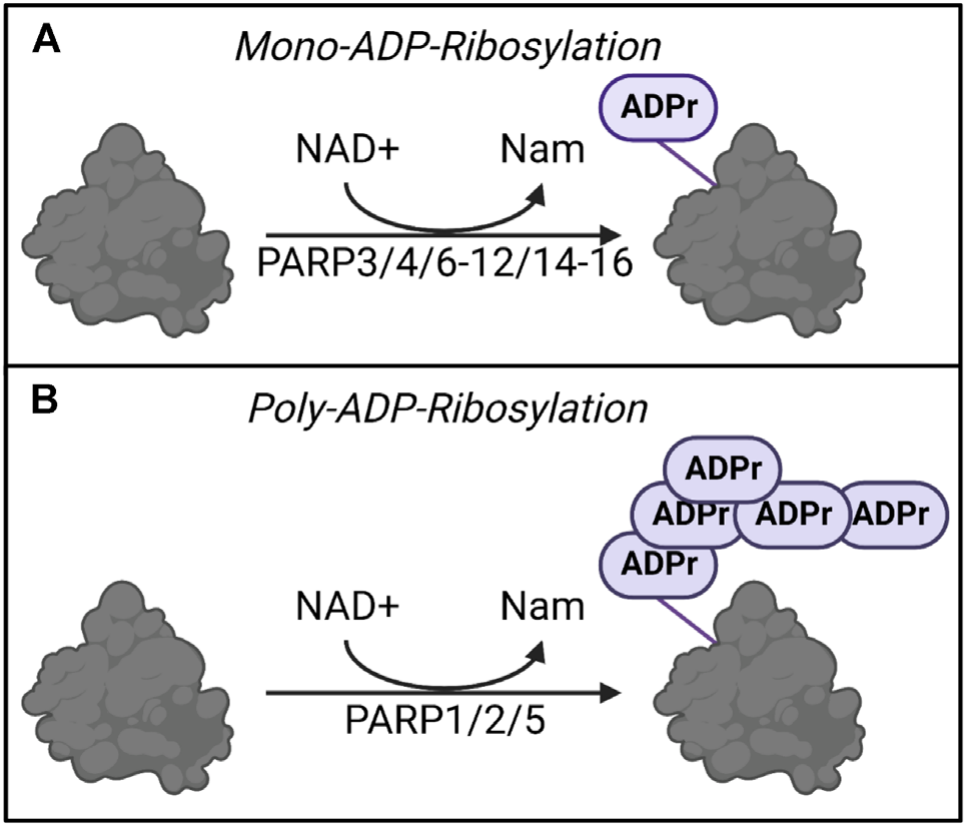
Schematic representation of the ADP-ribosylation reaction. Mono-ADP-ribosylation implies the transfer of a single ADP-ribose moiety onto the substrate, while poly-ADP-ribosylation involves the catalysis of poly-ADP-ribose chains. The PARP enzymes involved in each of the two reactions are also indicated. Created with Biorender.com

Post-translational modifications are chemical alterations essential for cell physiology (1,2). There are over 300 known post-translational modifications including phosphorylation, ADP-ribosylation, ubiquitylation and sumoylation (2), which help increase proteomic diversity. ADP-ribosylation involves the transfer of one or more ADP-ribose (ADPr) moieties from nicotinamide adenine dinucleotide (NAD+) to target proteins (1,3,4). NAD+ is a pyridine nucleotide co-factor, vital for maintenance of cellular homeostasis (5). There are two types of ADP-ribosylation depending on the number of ADP-ribose moieties transferred. The transfer of a single unit of ADP-ribose to target proteins is known as mono-ADP-ribosylation, or MARylation, while the transfer of multiple units of ADP-ribose to target proteins is known as poly-ADP-ribosylation or PARylation (Figure 1) (3,5–8). Enzymes catalyzing this reaction belong to the ADP-ribosyltransferases (ARTs) superfamily, which includes members of the poly (ADP-ribose)-polymerase (PARP) family of proteins (1–3,9). ADP-ribosylation is involved in multiple processes important for cell physiology including proliferation, cell motility and transcription, but PARP proteins are best characterized for their role in DNA damage repair (1,4,10).

First identified in 1963 by Chambon *et al.* (11), the PARP superfamily of proteins is now made up of 17 unique members that vary widely in terms of size, structure and functions (3,7,12). All members of this family (with the exception of PARP13) contain a C-terminal PARP catalytic domain, which is involved in ADP-ribosylation of target proteins which can include PARP proteins themselves (1,8,13). Enzymatically, the difference between MARylating and PARylating enzymes is generally thought to lie within the ‘catalytic triad sequence’ in the PARP domain, with the H–Y–E sequence being specific to PARylating enzymes, and the H–Y–I/L/Y sequence being present in MARylating enzymes (7,10).

In general, just like the case with most other post-translational modifications, ADP-ribosylation of substrate proteins, be it MARylation or PARylation, provides a new interaction surface on the substrate and thus alters its binding partners. Indeed, a wide variety of MAR/PAR-binding domains has been described (7,14–18).

As compared to other post-translational modifications, ADP-ribosylation is more difficult to study biochemically since these modifications are heterogeneous and bulky, and can occur at multiple residues including serine, tyrosine and lysine for PARylation and arginine, cysteine and histidine for MARylation (5,19). Additionally, the presence of multiple active PARPs in cells at any given time makes it complicated to determine contributions made by specific PARP proteins (19). Nevertheless, recent proteomic studies have identified hundreds of ADP-ribosylated and ADP-ribose-binding proteins (14,17,20–25).

The major contributor to our knowledge on ADP-ribosylation are studies done on PARP1 and PARP2, particularly studies focused on their roles in maintenance of genomic stability. The anti-PAR monoclonal antibody 10H which binds to PAR chains consisting of >10 ADP-ribose moieties has been extensively used to study PARP1 and PARP2 (26). On the other hand, MARylation remains understudied due to the lack of specific antibodies (26,27). An alternate way to study MARylation is by using ADP-ribose-binding domains (19). The macrodomain from the archaebacterial protein af1521 fused to a GST tag can be used to detect ADP-ribosylated proteins (28,29). More recently, Garcia-Saura *et al.* (30) described a version of this tool called MacroGreen. MacroGreen has been modified to have higher affinity binding to ADP-ribosylated targets as well as decreased ADP-ribosyl glycohydrolase activity. While these tools in general do not accurately distinguish MARylation from PARylation, specific MAR-binding peptides have also been recently developed based on the MAR-binding macrodomains of PARP14 (7,31).

### PARP10 and PARP14 as members of the PARP superfamily of proteins

**Figure 2.**
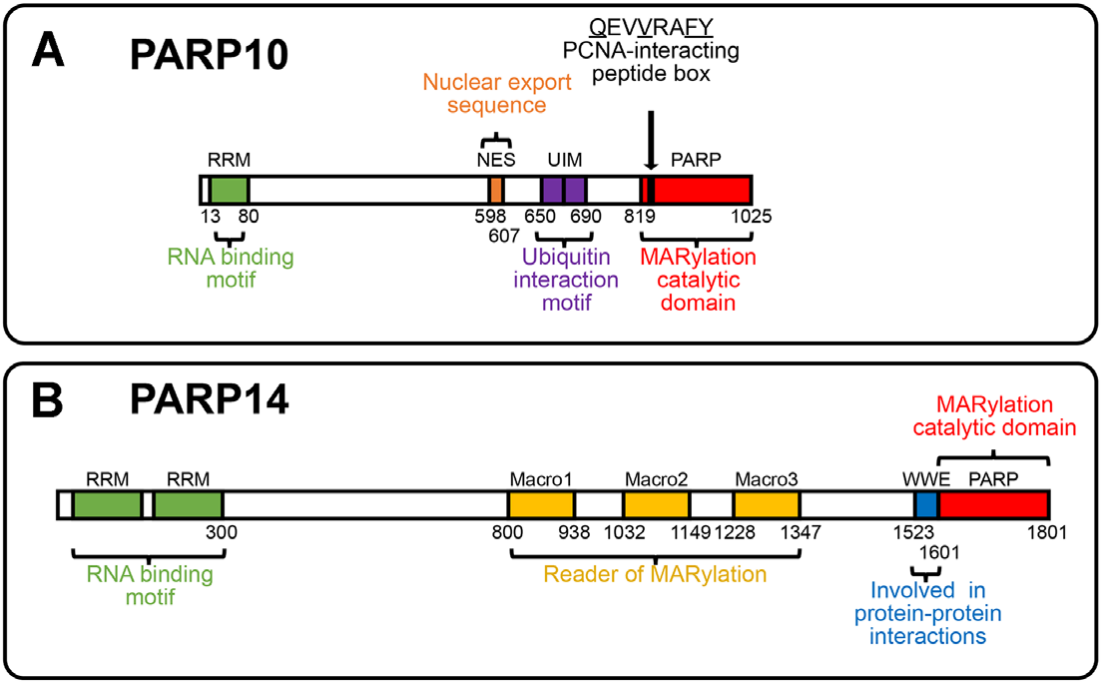
Schematic representation of PARP10 and PARP14. PARP10 and PARP14 both contain RNA recognition motifs (RRM) domains and a PARP catalytic domain, which catalyzes mono-ADP-ribosylation. In addition, PARP10 also has a nuclear export sequence (NES) and two ubiquitin-interacting motifs (UIM). The PIP-box at position 834 is highlighted and the sequence is shown, with conserved amino acids underlined. PARP14 is further classified in the macro sub-family due to the presence of three macro domains which bind to mono-ADP-ribose, making it both a reader and modifier of MARylation. PARP14 also contains a WWE domain, which is important for protein interactions.

PARP1 and PARP2 are the most well studied members of this family and have been shown to be indispensable for DNA damage repair (3). They were hence highly sought-after targets for therapeutic agents and in fact PARP1 inhibitors have been used successful in the clinic for treating ovarian and breast cancers (3,32). As compared to PARP1 and PARP2, the other members of the PARP family are not as well studied. However, they have gained more attention over the past years due to increasing amount of data showing a role for them in multiple processes and pathways. This review will specifically focus on PARP10, also known as ARTD10 and PARP14, also known as ARTD8 (Figure 2).

PARP10 was the first mono-ADP-ribosyl transferase characterized (33,34). PARP10 was initially identified as a MYC-interacting protein and was shown to suppress replication of certain RNA viruses via inhibition of NF-κB (33,35). However subsequent studies (35–37) showed that PARP10 is also involved in DNA damage repair. PARP10 consists of 1025 amino acids and encompasses an RNA recognition motif (RRM) domain, a nuclear export sequence (NES), two ubiquitin-interacting motifs (UIM) and the PARP catalytic domain (Figure 2A) (34,36). The NES is leucine rich and promotes localization to cytoplasm and the UIMs regulate nuclear transport of protein (33). PARP10 also contains a PCNA-interacting peptide box (PIP-box), which is unique to PARP10 and not present in other members of the PARP family (36). The PARP domain is involved in MARylation of target proteins and multiple potential targets of PARP10 have been identified (38). Additionally, several PARP10 inhibitors have also been developed (39).

Like PARP10, PARP14 is also a MARylating protein. PARP14 has been shown to be involved in multiple cellular processes including DNA repair, inflammatory signaling, innate immunity and transcriptional control (2,40). It consists of 1801 amino acids and is the largest member of this family of proteins (Figure 2B) (3,27). It comprises two RRM domains, three macro domains, a WWE domain and a PARP catalytic domain (Figure 2B) (27,41). The RRM domains binds to RNA but if there are specific targets is still unknown (41). The presence of two or more RRM domains allows the formation of a larger binding platform, which enables binding to nucleotide sequences of 8–10 nucleotides (42). Interestingly, RRM domains, which are also present in PARP10, have also been proposed to bind to PAR chains (16,43). While it is not known if the RRM domains in PARP14 (or those in PARP10) do bind PAR chains, there presence may suggest a potential cross-talk between MARylating enzymes and PARP1. The presence of the macro domains categorizes PARP14 to be in the macro sub-family, which also includes PARP9 and PARP15. This subgroup is unique in its ability to specifically bind ADP-ribose through the macro domains (27,38). The WWE domains of PARP14 contain a Trp-Trp-Glu region (2,41). These domains were shown to be involved in protein-protein interactions (27), via stabilization of PARP14-protein structure (2). Finally, the PARP catalytic domain is involved in MARylation of target proteins, which can include PARP14 itself (27,44). PARP14 is thus able to both read and catalyze MARylation via its macro and PARP domains respectively (27,45).

Unbiased identification of specific PARP10 and PARP14 substrates is of significant importance. Using protein microarrays, Feijs *et al.* identified 78 potential PARP10 substrates and 142 potential PARP14 substrates (46). More recently, proteomic-based studies using different approaches have been conducted to unbiasedly identify the substrates of MARylating proteins from cells. In a recent study by Saei *et al.* (47), a novel method called System-wide Identification of Enzyme Substrates by Thermal Analysis (SIESTA) was utilized as an unbiased approach to identify potential PARP10 substrates. SIESTA works by identifying thermal stability changes upon the post-translational modification of substrates. This study identified 58 proteins as potential PARP10 targets with the majority of them being novel ones. Carter-O’Connell *et al.* used a designed PARP14 variant combined with a BioID approach for proximity-dependent labeling to identify PARP14-specific MARylation targets. A total of 114 potential substrates were identified using this approach, including multiple DNA repair proteins such as MRE11, KU70, KU80 and RAD50.

## EMERGING ROLES OF MONO-ADP-RIBOSYLATION IN DNA REPAIR

The hallmarks of cancers, first defined by Hanahan and Weinberg (48), represent a set of characteristics different types of cancer cells share. The updated list consists of ten traits, including genome instability and mutations (49). Importantly, these hallmarks of cancer, including DNA repair deficiency, can be targeted to enhance response to various anticancer therapies (49,50). Indeed, PARP1 inhibitors have been used successful in the clinic for treating ovarian and breast cancers (3,32). Here, we outline some of the key roles of PARP10 and PARP14 in modulating DNA damage repair, and how PARP10/PARP14 status affects cancer development and the response to chemotherapeutics.

### Roles of PARP10 in genomic stability and carcinogenesis

PARP10 has first been shown to play a role in DNA damage repair in Nicolae *et al.* (36). In this study, the authors show that PARP10 promotes translation synthesis (TLS), by binding to ubiquitinated proliferating cell nuclear antigen (PCNA). TLS is a DNA damage tolerance pathway that allows the bypass of lesions (51). PCNA is the key regulator of TLS and its role is regulated by post-translational modifications, in particular ubiquitination and sumoylation (52,53). PCNA mediates the recruitment of specialized TLS polymerases (51,54). This switch between normal DNA replication high fidelity polymerases and TLS-specific low fidelity polymerases enables the eventual bypass of damage sites (51,53,54). This is tightly regulated to prevent accumulation of mutagenesis (53).

**Figure 3.**
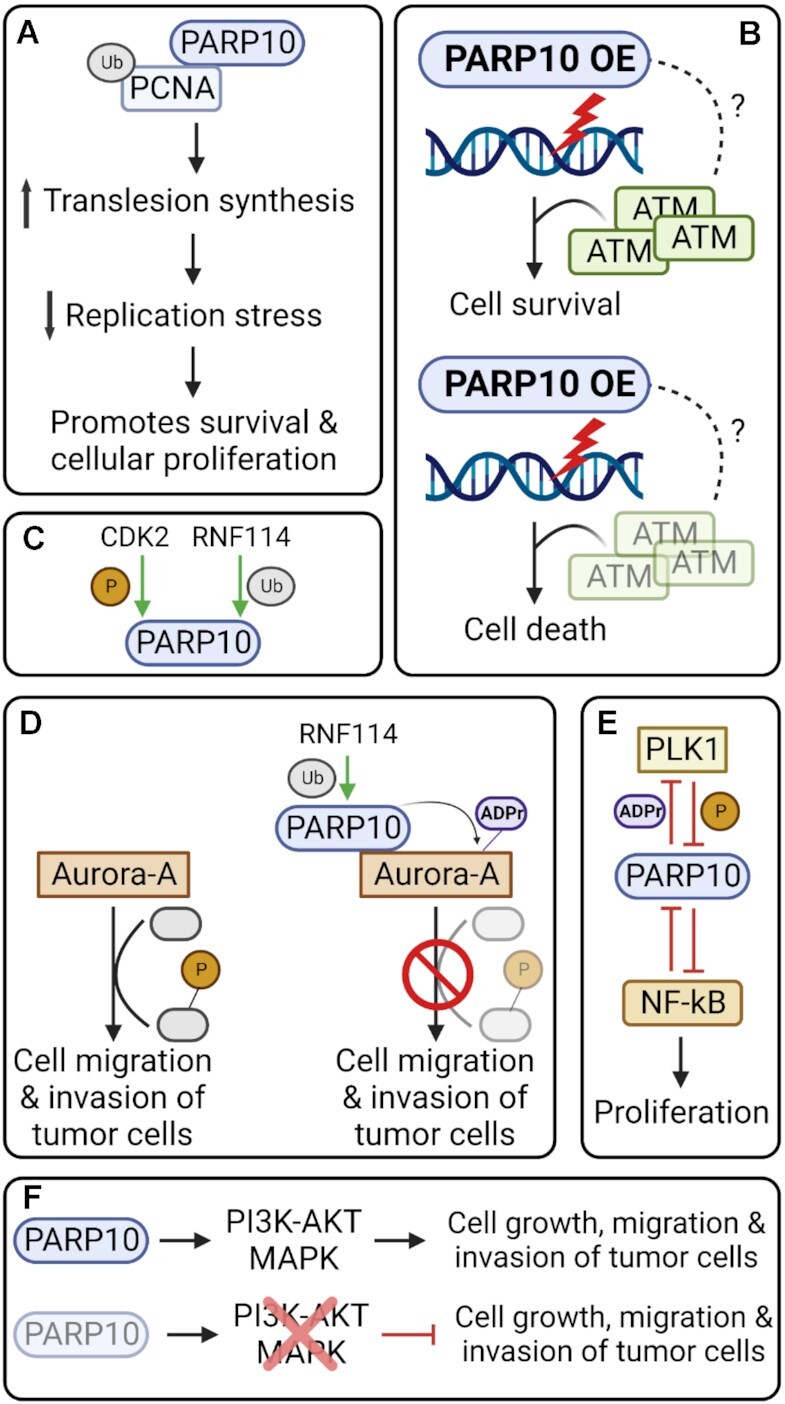
PARP10 roles in genome stability and cellular proliferation. (**A**) PARP10 binds to ubiquitinated PCNA to enhance PCNA-mediated translation synthesis (TLS), decreasing replication stress and promoting cell survival. PARP10 overexpression, observed in various cancers, confers protection against increased replication stress associated with carcinogenesis by increasing TLS. (**B**) PARP10-overexpressing cells are reliant on ATM for survival. PARP10 affects recruitment of ATM to nascent DNA upon replication stress. Loss of ATM in PARP10-overexpressing cells leads to cell death. (**C**) Upstream modulators of PARP10 include CDK2 and RNF114. PARP10 is phosphorylated by CDK2 to regulate cell cycle progression. RNF114 ubiquitinates PARP10 through K27-linked polyubiquitination, enhancing PARP10 enzymatic activity. (**D**) PARP10 MARylates several kinases involved in cell cycle regulation including Aurora A. MARylation by PARP10 suppresses the kinase activity of Aurora A, hindering its ability to promote tumor proliferation and metastasis. (**E**) PARP10 phosphorylation by PLK1 abrogates PARP10-mediated inhibition of the NF-κB pathway. PARP10 is also able to MARylate PLK1 leading to a decrease in its kinase activity, suggesting a PARP10/PLK1/NF-κB feedback loop. (**F**) PARP10 has also been shown to be involved in regulation of the PI3K-AKT and MAPK signaling pathways to promote cell growth, migration and invasion. The exact mechanism is not understood yet. Created with Biorender.com

Nicolae *et al.* demonstrated that PARP10 protects replication forks against fork stalling agents such as hydroxyurea (HU) and ultraviolet light (UV). More specifically PARP10 promotes the bypass of DNA lesions that accumulated as a result of HU or UV treatment. PARP10 interacts with ubiquitinated PCNA via its PIP-box and UIM motifs (Figure 3A). The PARP10-PCNA interaction promotes the recruitment of TLS polymerases to stalled replication forks, resulting in DNA lesion bypass and alleviating replication stress. Overall, this study showed that the MARylation activity of PARP10 is important to promote genomic stability via PCNA.

A subsequent study by Schleicher *et al.* (37) also showed a role for PARP10 in alleviating replication stress. In this case a novel role for PARP10 in promoting cellular proliferation was uncovered. The authors also probed datasets to show that PARP10 is amplified in a variety of cancers, and hence a role as a putative oncogene was hypothesized. In this study, PARP10 overexpression was demonstrated to promote both *in vitro* and *in vivo* cellular proliferation. PARP10 overexpression allowed restart of stalled replication forks, alleviating replication stress and promoting cellular proliferation. It is possible that this is partially via its role in TLS, but this needs further investigation. Additionally, PARP10 overexpression in the non-transformed cell line RPE-1 resulted in significant tumor growth in xenograft mouse studies, suggesting oncogene like properties. Moreover, using a deletion fragment it was also shown that the catalytic domain of PARP10 is required for these observations. The authors speculate that PCNA MARylation by PARP10 leads to an increase in ubiquitinated PCNA. This would in turn lead to an increase in TLS polymerase recruitment, alleviating stress and promoting proliferation.

More recently in 2022, the same group utilized genome-wide CRISPR knockout screens to identify genes required for viability of PARP10-overepressing cells (55). ALKBH2, PRDM10 and ATM were identified and validated as top hits. ATM was further investigated given its central role in DNA damage and repair. Loss of ATM reduced proliferation of multiple PARP10-overexpressing cell lines. It was further found that under conditions of high replications stress, ATM recruitment to stressed replication forks is enhanced in PARP10-overexpressing cells. This implies a potential role for PARP10 in regulating ATM recruitment and binding to nascent DNA (Figure 3B). Whether this is dependent on PARP10 MARylation activity is not known.

A study by Zhao *et al.* (35) in 2018 identified another role for PARP10 in tumorigenesis via its MARylation activity. In this study, PARP10 deficiency was shown to promote tumor cell migration and invasion via MARylation of the Aurora A protein. Aurora A is serine-threonine protein kinase that plays a role in tumorigenesis (56). It is often amplified in cancers (35), and is involved in cell cycle as well as tumor invasion and metastasis via epithelial–mesenchymal transition (EMT) (56). PARP10 interacts with and MARylates Aurora A, inhibiting its kinase activity and any downstream signal. PARP10-Aurora A interaction did not impact its role in cell cycle, but it did regulate its role in EMT, suggesting a role for PARP10 in mediating migration and invasion of tumor cells. More recently, Zhao *et al.* (57) identified RNF114 as a novel regulator of PARP10. The MARylation activity of PARP10 is enhanced by RNF114-mediated ubiquitination. Subsequent MARylation of Aurora A by ubiquitinated PARP10 suppressed its kinase activity and downstream signaling, negatively impacting tumor metastasis.

A study by Di Paola *et al.* (58) in 2022 further explored the relationship between PARP10 and Aurora A. It had previously been shown that PARP10 is phosphorylated by CDK2 to support cellular growth and proliferation (59). Here, the authors showed that PARP10 is necessary to prevent defects in the G2/M phase progression and this is dependent on its ability to MARylate Aurora A. MARylation of Aurora A by PARP10 activates its kinase activity, promoting recruitment to centrosomes and ensuring proper G2/M phase progression (Figure 3C, D). This further demonstrates the importance of PARP10 in promoting cellular proliferation.

In 2020, Tian *et al.* (60) uncovered a role for PARP10 in hepatocellular carcinoma (HCC) progression via polo-like kinase 1 (PLK1) activity and NF-κB signaling. PLK1 is often overexpressed in tumors and its expression levels play a role in progression of various cancers including HCC. In this study it was demonstrated that PLK1 directly interacts with PARP10 both *in vitro* and *in vivo*. PLK1 also phosphorylates PARP10 which leads to the activation of NF-κB signaling. It was proposed that PARP10 is a direct target of NFκB and indeed NF-κB binds to the PARP10 promoter to suppress its transcription, resulting in a negative feedback loop. Moreover, PLK1 is MARylated by PARP10 both *in* vitro and *in vivo*, leading to a decrease in its kinase activity. Overall, these results suggest a novel role for PARP10 in modulating HCC progression via the PARP10/PLK1/NF-κB feedback loop. Here two different post-translational modifications are important – phosphorylation of PARP10 by PLK1 and MARylation of PLK1 by PARP10 (Figure 3E). The study also briefly assessed PLK1 and NF-κB inhibitors in suppressing HCC progression. Combination treatment with PLK1 and NF-κB inhibitors led to a decrease in HCC growth and metastasis. PARP10 inhibitors were not assessed but would be worthwhile investigating.

More recently, Zhou *et al.* (61) examined the function of PARP10 in oral squamous cell carcinoma (OSCC), the most common type of head and neck cancer. Using data from patient cohorts, they showed that PARP10 is upregulated in OSCC and higher level is associated with a poorer prognosis. Mechanistically, the authors uncovered a novel role for PARP10 in mediating the PI3K-AKT and MAPK signaling pathways (Figure 3F). PARP10 depletion inhibits OSCC cell growth and invasion by impairing those signaling pathways. Further investigation is needed to mechanistically understand how PARP10 affects these signaling pathways and if its MARylation activity plays a role.

### Roles of PARP14 in immune and metabolic signaling

**Figure 4.**
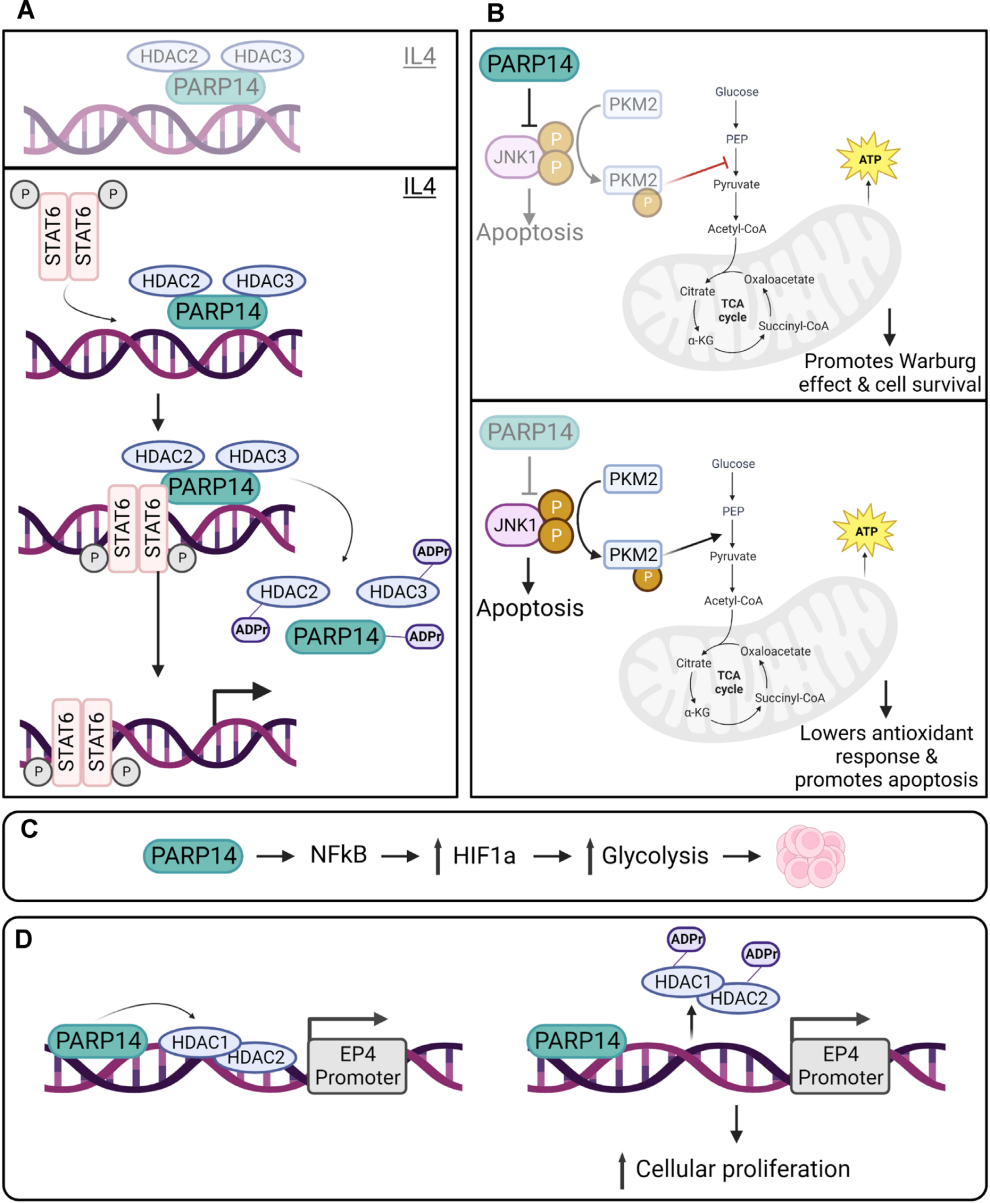
PARP14 cell signaling activities. (**A**) PARP14 plays a central role in IL-4 dependent gene transcription. In the absence of IL-4, PARP14 forms a complex with HDAC2 and HDAC3 and binds to gene promoter to silence transcription. In the presence of IL-4, STAT6 binds to the promoter region as well as PARP14 to activate its catalytic activity. MARylation of the PARP14-HDAC2-HDAC3 complex by PARP14 triggers its dissociation, activating gene transcription. (**B**) The PARP14-JNK1-PKM2 axis plays a central role in regulating the Warburg effect. PARP14 can interfere with the kinase activity of JNK1, an upstream regulator of PKM2. Inhibition of JNK1 maintains low activity of PKM2 and promotes aerobic glycolysis. In the absence of PARP14, JNK1 kinase activity is no longer restricted. Glucose conversion to pyruvate is enhanced, leading to lower antioxidant response and increase in apoptosis. (**C**) PARP14 promotes cell proliferation by modulating the HIF-1α level via the NF-κB pathway in acute myeloid leukemia cells. (**D**) MARylation activity of PARP14 is essential to promote EP4 expression level in colorectal cancer cells. HDAC1 and HDAC2 bind to the EP4 promoter region to inhibit its expression. MARylation by PARP14 promote their dissociation, allowing expression of EP4. Created with Biorender.com

PARP14 was first characterized as a regulator of Signal Transducer and Activator of Transcription 6 (STAT6) (27). The macrodomains of PARP14 enhance the production of the cytokine interleukin 4 (IL-4) via STAT6 (27,62). In the absence of IL-4, PARP14 forms a complex with HDAC2 and HDAC3 to inhibit gene transcription. Upon IL-4 stimulation, activated STAT6 binds to target genes as well as PARP14 and promotes the catalytic activity of PARP14. Subsequently PARP14 MARylates itself, HDAC2 and HDAC3, promoting dissociation of the complex from promoters, including the IL-4 promoter to allow gene transcription (Figure 4A). IL-4 has conflicting roles in cancer—some studies show that it is a strong anti-tumor therapy agent while others demonstrate that it promotes tumor progression (63). It was suggested that PARP14 performs different roles in tumor development and progression of different cancer types (27). Given the role of STAT6 in B-cell survival, PARP14 is also implicated in promoting B-cell specific tumorigenesis such as diffuse large B-cell lymphoma and multiple myeloma (27,39).

In 2013, Barbarulo *et al.* (64) investigated the role of PARP14 in the proliferation of multiple myeloma, a B-cell malignancy given its role in B-cell survival. This study demonstrated a role for PARP14 in promoting survival of multiple myeloma cells via Jun N-terminal kinase (JNK) signaling (Figure 4B). JNK1 and JNK2 are often considered redundant but have also been shown to have tissue specific functions. The results of this study indicate that PARP14 promotes JNK2 dependent survival of multiple myeloma cells by inhibiting JNK1. PARP14 binds to JNK1 via its C-terminal domain, resulting in inhibition of JNK1-mediated apoptosis. Additionally, inhibiting PARP14 using the specific inhibitor PJ-34 sensitized multiple myeloma cells to therapeutic agents such as dexamethasone and bortezomib. However, it should be noted that JNK2’s role as an oncogene is limited to terminally differentiated B-cells, such as those in multiple myeloma. Hence these findings would only have clinical implication for patients with multiple myeloma.

Subsequently, Iansante *et al.* (65) found a role for PARP14-JNK1 in regulating the Warburg effect by modulating the activity of the pyruvate kinase M2 isoform (PKM2). PKM2 is a regulator of the Warburg effect, also known as aerobic glycolysis. This study aimed at determining how the Warburg effect helped tumor cells evade apoptosis. This was investigated specifically in hepatocellular carcinoma (HCC) cells, which is one of the cancers known to have increased aerobic glycolysis. Results from this study show that PARP14 inactivates JNK1, which in turn activates PKM2 via phosphorylation. JNK1 normally phosphorylates PKM2 at the Thr365 residue, which increases PKM2 activity. Low PKM2 activity enhances tumor survival by promoting the Warburg effect while high PKM2 activity promotes apoptosis. Overall, this suggests a role for PARP14-JNK1-PKM2 axis in regulating the Warburg effect in HCC cells.

Additionally, Zhu *et al.* (66) examined the role of PARP14 in glycolysis in acute myeloid leukemia (AML). PARP14 was found to be upregulated in AML and higher levels were associated with overall reduced patient survival. This study found that PARP14 enhances AML cell proliferation by promoting glycolysis. This function is dependent on PARP14’s ability to modulate HIF-1α expression via the NF-ƙB pathway (Figure 4C). HIF-1α (hypoxia inducible factor 1 subunit alpha) is a protein involved in angiogenesis and cellular metabolism. This study demonstrates a novel role for PARP14 in regulating the NF-ƙB/HIF-1α axis, however other pathways might also be involved in regulating this axis.

In a 2022 study, Mashimo *et al.* (67) examined the function of PARP14 in human colorectal cancer and showed that PARP14 modulates expression of EP4 receptors (Figure 4D). EP4 plays a key role in colorectal cancer. This study showed that MARylation of HDAC1 and HDAC2 by PARP14 is required to induce expression of EP4 receptors in human colon cancer cells. MARylation of HDAC1 and HDAC2 prompts their dissociation from the promoter, allowing for an increase in EP4 receptor mRNA expression level. Inhibition of PARP14 abrogates activation of the EP4 receptor and reduces proliferation of colon cancer cells, making PARP14 a good target for colon cancer therapy.

### Roles of PARP14 in DNA repair and genome stability

In addition to its role in immune signaling and metabolism, multiple studies have demonstrated that PARP14 is involved in maintenance of genomic stability via several mechanisms. Two of the key factors involved in maintenance of genomic stability are the Breast Cancer Susceptibility Genes BRCA1 and BRCA2, whose function is to mediate the loading of RAD51 to single-stranded DNA at DNA damage sites (68). The BRCA proteins are key players in homologous recombination (HR), the error free pathway by which DNA double stranded breaks are repaired (68,69). BRCA1/2 also have independent roles in stabilization of stalled replication forks which can occur under conditions of high replication stress (70,71). BRCA1 or BRCA2 mutations significantly increase the likelihood of developing breast and ovarian cancer (68). It was demonstrated that PARP14 depletion lowers HR efficiency via the DR-GFP reporter assay (44). Results from this study indicate that PARP14 interacts with and potentially MARylates RAD51. RAD51 loading onto ssDNA and subsequent unloading is regulated by BRCA proteins and both are crucial steps during HR (69). Interference with either the loading or unloading of RAD51 impacts HR efficiency and promotes accumulation of genomic instability. This study proposed that RAD51 MARylation by PARP14 helps facilitate unloading of RAD51. More specifically the Macro2 domain of PARP14, which recognizes MARylation on substrates, was shown to bind RAD51 following DNA damage. Additionally, overexpression of Macro2 inhibits HR, implying a dominant negative effect. The results suggest that MARylation of RAD51 by PARP14 (and potentially other enzymes) is needed for efficient HR. Hence depletion of PARP14 results in more persistent RAD51 foci, preventing complete and efficient HR. This indicates that PARP14 functions in HR following RAD51 foci formation, and facilitates its removal. RAD51 MARylation by PARP14 might thus be important for strand invasion, or for the subsequent removal of RAD51 from the stable displacement loop formed upon strand invasion. Further investigation is needed to better understand this connection between PARP14 and RAD51 in HR.

**Figure 5.**
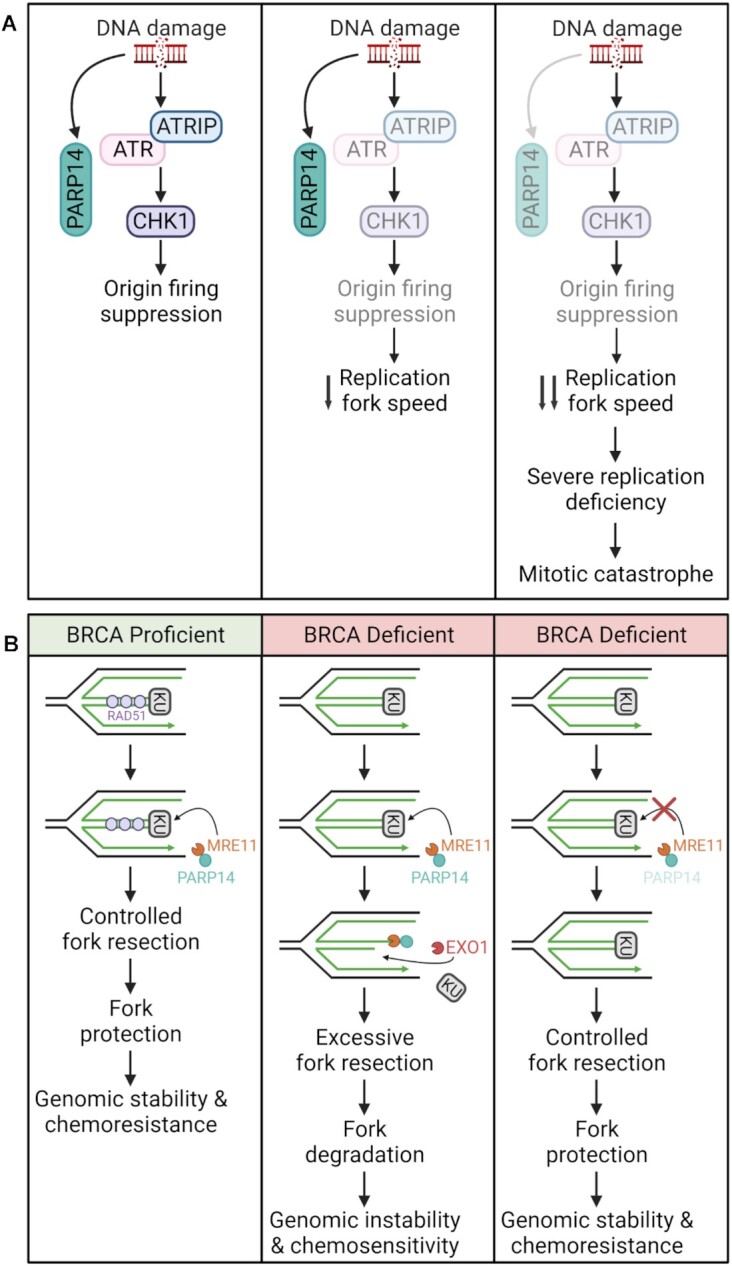
PARP14 roles in DNA damage repair. (**A**) Activation of the ATR-CHK1 pathway prevents origin firing in cells with DNA damage. ATR inhibition results in a decrease in replication fork speed. In PARP14-deficient cells, the decrease in replication fork speed is more significant leading to severe replication deficiency, and eventually leading to mitotic catastrophe. (**B**) In BRCA1/2 deficient cells, RAD51 loading is compromised at stalled replication forks, which results in excessive degradation by nucleases MRE11 and EXO1. MRE11 has been shown to be recruited as a complex with PARP14 to KU-bound stalled replication forks. MRE11-mediated resection causes removal of KU, allowing EXO1 access to the DNA. Processing by MRE11 and EXO1 leads to excessive fork degradation and genomic instability. Inhibition of PARP14 prevents initial MRE11-mediated resection and the subsequent EXO1-mediate resection, protecting stalled replication forks and promoting genomic stability. Created with Biorender.com

Given that these findings indicated that PARP14 affects replication stress and could impact how tumors respond to genotoxic drugs, a subsequent study utilized CRISPR screening technology to identify synthetic lethal interactors of PARP14 (72). A genome wide synthetic lethality CRISPR screen was performed using the Brunello human CRISPR knockout lentiviral-based library. This library covers 19114 genes with four different guide RNAs on average. Two different computational algorithms, RSA and MAGeCK, were used to create a list of genes that were lost in PARP14-knockout cells but not wild-type cells. The ATR-CHK1 pathway was identified as essential for viability of cells depleted of PARP14. Multiple components of the ATR-CHK1 pathway were identified as top hits, including CHK1, TOPBP1 and MRE11. It was further shown that ATR pathway inhibition in PARP14 deficient cells results in defective replication and cell cycle checkpoint failure. Consequently, cells with incompletely replicated DNA enter mitosis resulting in mitotic catastrophe (Figure 5A). It was also demonstrated that PARP14 deficient cells are hypersensitive to ATR-CHK1 pathway inhibitors, including VE822 (ATRi) and rabusertib (CHK1i).

Besides its role in DNA damage repair PARP14 is also implicated in cell cycle regulation. In a study by O’Connor *et al.* (73), it was shown that PARP14 promotes G1/S phase transition in specific cell lines via regulation of the retinoblastoma (RB) pathway. Cyclin D1 phosphorylates Rb protein, which is then no longer able to repress the transcription of genes responsible for transition from G1 to S phase (74). This study showed that PARP14 promotes G1/S phase transition in certain cell lines by modulating cyclin D1 level. This phenotype was observed in both non transformed epithelial and cancer cells, including RPE-1, HCT116, MCF7 and HCC1395. This was not observed in cell lines with impaired RB pathway such as HeLa. More specifically PARP14 promotes phosphorylation of Rb protein by regulating cyclin D1 levels via its 3’ UTR. Since the RB pathway is a tumor suppressor pathway (75), this suggests a role for PARP14 in modulating tumor progression.

More recently, a novel role for PARP14 in modulating fork stability specifically in BRCA1/2 deficient cells was uncovered (76) (Figure 5B). BRCA proteins protect nascent DNA from excessive resection by nucleases such as MRE11 and EXO1 at stalled replication forks (71,77). It was shown that PARP14 interacts with MRE11 and loss of PARP14 at stalled replication forks suppresses MRE11-mediated degradation in BRCA-deficient cells. Using both a catalytically inactive mutant and two novel inhibitors, it was further demonstrated that this occurs via the MARylation activity of PARP14. Finally, the authors showed that the KU complex promotes the recruitment of the PARP14-MRE11 complex. This allows initial resection by MRE11 and subsequent long-range resection by EXO1, resulting in fork degradation.

## DEVELOPMENT OF INHIBITORS OF PARP10 AND PARP14

PARP1 inhibitors, which inhibit PARP1 and PARP2, are FDA approved and widely used in clinics for patients with BRCA1 and BRCA2 mutations (5). However, there is still a lack of selective inhibitors for the other members of the PARP family, in particular the mono-ADP-ribosyltransferases. This is partially because most PARP inhibitors are designed to compete with the nicotinamide moiety in NAD+ and the NAD+ binding pocket is highly conserved among the PARP family members, making it hard to obtain highly selective inhibitors (7,78). Given their recently discovered roles in cancer development and response to chemotherapeutics, PARP10 and PARP14 represent promising clinical targets. Here, we highlight a few compounds that have been characterized over the past several years as PARP10 (Table 1) or PARP14 (Table 2) inhibitors.

**Table 1.** Inhibitors of PARP10

Drug	References	IC50 (μM)	Selectivity
Compound 19	Ekblad *et al.*, 2015	2.0	>20-fold over PARP1/10/15
8b	Holecheck *et al.*, 2018	0.4	Selective over PARP1
8r	Holecheck *et al.*, 2018	0.39	Selective over PARP1
OUL35	Venkannagari *et al.*, 2016	0.329	>12-fold selective against all members except PARP11
Compound 22	Morgan *et al.*, 2019	1.8	Selective for PARP10

**Table 2.** Inhibitors of PARP14

Drug	References	IC50 (μM)	Selectivity
H10	Peng *et al.*, 2017	0.49	∼24-fold over all other members
4t	Upton *et al.*, 2017	0.16	∼6-fold against PARP5/10
Compound 1	Yoneyama-Hirozane *et al.*, 2017	0.58	>30-fold against PARP1
Compound 2	Yoneyama-Hirozane *et al.*, 2017	0.31	>26-fold against PARP1
Compound 8	Wang *et al.*, 2014	1.69	-
8k	Holecheck *et al.*, 2018	0.78	Selective over PARP1
8m	Holecheck *et al.*, 2018	0.7	Selective over PARP1
RBN012759	Schenkel *et al.*, 2021	0.0003	>300-fold selective over all PARP family members

Despite having strong link to various pathogenic states such as inflammatory diseases and cancer, PARP10 and PARP14 inhibitors have not been well characterized. Most inhibitors previously identified (78–86) have half-maximal inhibitory concentrations in the micromolar range and target the catalytic domain, and do not appear to share common chemical groups. Unfortunately, most of them are only selective against a few members, making them not ideal candidates. However, there are two novel PARP14 inhibitors that exhibit selectivity over all the members of the PARP family—H10 and RBN012759. H10 binds both the NAD+ and adenine subsites while RBN012759 binds only to the NAD+ pocket.

H10 was identified by Peng *et al.* (78) using a high throughput microarray-based strategy. It has an IC50 of about 490 nm and is about 24-fold selective over all other PARP family members. H10 not only binds to the nicotinamide binding site of PARP14 but also to the adenine binding site, helping with decreasing non-selective binding. Results show that H10 inhibits endogenous PARP14 activities and activates JNK1 phosphorylation, in line with previous findings (64,65). RBN012759 was discovered more recently by Schenkel *et al.* (86). It exhibits over 300-fold selectivity over all other members of the PARP family and can be utilized both *in vitro* and *in vivo*. This inhibitor was characterized in primary macrophages stimulated with interferon-gamma. PARP14 inactivation in macrophages promotes the pro-inflammatory phenotype of macrophages which stimulates anti-tumor response (27,64). RBN012759 was shown to inhibit MARylation in a dose-dependent manner and increase the anti-tumor inflammatory phenotype of macrophages.

Because of its roles described above in immunity and the DNA damage response, we speculate that PARP14 inhibitors can potentially be employed to regulate inflammation and as anti-cancer agents. Because of its role in HR, PARP14 inhibitors could be employed as chemo/radio-sensitizers. Moreover, Dhoonmoon *et al.* (72) demonstrated that PARP14 inhibition sensitizes cells to CHK1 and ATR inhibitors, both currently in clinical trials. Regarding PARP10, its established role in cellular proliferation and carcinogenesis may suggest that PARP10 inhibitors could also be employed in cancer treatment, particularly breast and ovarian cancers, since PARP10 is specifically overexpressed in those tumors (37).

In conclusion, there are currently several PARP10 and PARP14 inhibitors that have been developed but need to be further characterized. These inhibitors will not only allow mechanistic studies but will also help assess PARP10 and PARP14 as therapeutic targets. Additionally, non-selective binding of these inhibitors against all other members of the PARP family needs to be further explored.

## CONCLUSIONS AND FUTURE DIRECTIONS

PARP1 and PARP2 have been extensively studied and well characterized for their role in DNA damage repair and cancer progression. Additionally, anti-cancer therapeutics targeting PARP1 and PARP2 have been developed and are currently widely used in clinics. However, much less is known about the other members of the PARP family of proteins, especially the MARylating members. Here, we highlighted the roles of MARylation, and in particular those of PARP10 and PARP14, in DNA repair and cancer. A number of important open questions remain, and likely represent the focus of studies in the coming years. Chiefly among those is the identity of the relevant substrates of PARP10 and PARP14 in genome stability and cancer cell proliferation and tumorigenesis. Identifying how MARylation regulates these substrates, and the changes in binding partners induced by the modification, is likely to reveal critical mechanisms employed by PARP10 and PARP14 in cancer. In addition, the regulation of PARP10 and PARP14 activity in normal and cancer cells, as well as the mechanisms of their subcellular localization, particularly their recruitment to DNA, is of significant interest. Finally, since both PARP10 and PARP14 represent potential targets for cancer therapy, extensive characterization of PARP10 and PARP14 inhibitors in clinical settings is needed to determine therapeutic efficacy of these inhibitors.

## DATA AVAILABILITY

No new data were generated or analysed in support of this research.
